# Correlation between estradiol-to-testosterone ratio and thyroid peroxidase antibody positivity in men with treatment-naïve primary hypothyroidism or euthyroidism

**DOI:** 10.20945/2359-4292-2023-0256

**Published:** 2024-07-01

**Authors:** Saurav Shishir Agrawal, Anirban Sinha, Animesh Maiti, Partha Pratim Chakraborty, Asish Kumar Basu, Chhavi Agrawal, Biswabandhu Bankura

**Affiliations:** 1 Department of Endocrinology and Metabolism Medical College and Hospital Kolkata West Bengal India Department of Endocrinology and Metabolism, Medical College and Hospital, Kolkata, West Bengal, India; 2 Multidisciplinary Research Unit Medical College and Hospital Kolkata West Bengal India Multidisciplinary Research Unit, Medical College and Hospital, Kolkata, West Bengal, India

**Keywords:** Thyroid diseases, estradiol, testosterone, autoimmunity, hypothyroidism

## Abstract

**Objective:**

Thyroid diseases pose a substantial socioeconomic burden globally. The aim of this study was to evaluate the correlation between estradiol-to-testosterone (E2/T) ratio and thyroid peroxidase antibody (TPOAb) positivity in male patients with hypothyroidism or euthyroidism.

**Subjects and methods:**

Cross-sectional observational study including 115 male patients with hypothyroidism or euthyroidism. The patients were divided into two groups based on positive or negative TPOAb results, with TPOAb positivity defined by a serum TPOAb value ≥ 35 IU/mL.

**Results:**

Patients with positive TPOAbs, compared with those with negative TPOAbs, had a higher prevalence of goiter and obesity and higher levels of total cholesterol, triglycerides, and low-density lipoprotein (LDL) cholesterol. The median estradiol level was higher, and the median total testosterone and sex-hormone binding globulin (SHBG) levels were lower in the TPOAb-positive versus the TPOAb-negative group (p < 0.001). In subgroup analysis including only patients with hypothyroidism (n = 80), the median E2/T ratio was higher in the TPOAb-positive group (p = 0.016). The prevalence of TPOAb positivity increased with the increase in E2/T ratio quartiles, from 37.9% in the lowest quartile to 96.2% in the highest quartile (p value for trend across all quartiles < 0.001). On adjusted multivariate analysis, the E2/T ratio emerged as an independent predictor of TPOAb positivity. An E2/T ratio cutoff value of 6.565 x10-3 demonstrated the best diagnostic accuracy, with a sensitivity of 78.2% and specificity of 67.6%.

**Conclusion:**

The present study provides insights into the role of the E2/T ratio as a predictor of thyroid disorders.

## INTRODUCTION

Thyroid diseases impact 42 million individuals in India, with hypothyroidism prevailing among approximately 1 in 10 adults (1,2). Autoimmune thyroid disease (AITD), the most frequent endocrinopathy, manifests in the majority of individuals with thyroid disorders in iodine-depleted areas (3,4) and is characterized by circulating thyroid autoantibodies and infiltration of lymphocytes targeting thyroid antigens. Elevated serum thyroid peroxidase antibodies (TPOAbs) and antithyroglobulin antibodies (TgAbs) serve as clinical markers for the early detection of AITD and Hashimoto’s thyroiditis (5,6).

Among all autoimmune diseases, AITD is highly prevalent and has a high women-to-men ratio (7,8). Abnormal levels of sex hormones can provide a stage for genetic and environmental factors triggering AITD. Understanding the interaction and relationship between sex hormones and immune functioning, along with the potential pathological consequences of this interaction, may provide insights into the management of AITD.

The ratio of estradiol to total testosterone (E2/T) could help elucidate the relationship between sex hormones (*i.e.*, the synergistic effects of estrogen and androgen action) and clinical diseases, including autoimmune diseases (9). Although this topic has been rarely explored (10), a few studies from different countries have reported a correlation between sex hormone levels and AITD in men (11). However, robust data validating the correlation between thyroid autoimmunity and sex hormones remain scarce. Based on these considerations, the aim of this study was to evaluate the correlation between the E2/T ratio and TPOAb positivity in male patients with hypothyroidism or euthyroidism in India.

## SUBJECTS AND METHODS

This hospital-based, cross-sectional, observational study was conducted in a tertiary health care center from March 2018 through September 2019. The study included 115 consecutive male patients aged 18-60 years who presented with signs and symptoms of hypothyroidism. Patients on any form of hormone replacement therapy, with any other acute illnesses, with a history of systemic disease (chronic liver disease, chronic kidney disease, or cardiac failure) or other endocrinologic disorders (including hypogonadism), or with a prior history of thyroid surgery or treatment with radioiodine, levothyroxine, or antithyroid drugs were excluded.

The protocol of the study was approved by the Medical College Kolkata Institutional Ethics Committee (ECR/287/Inst /WB/2013; MC/KOL/IEC/NON-SPON/46/02-2018). Written informed consent was obtained from the patients or their relatives.

Patient demographics, clinical history, symptoms suggestive of hypothyroidism, and biochemical parameters were recorded.

The presence of AITD was determined by a serum TPOAb value ≥ 35 IU/mL, as validated in previous studies and according to the upper limit of normal in our laboratory (12,13). Normal values for thyroid-stimulating hormone (TSH) and free thyroxine (fT4) per laboratory standards were 0.4-4.5 μIU/mL and 0.8-1.7 ng/dL, respectively. Patients with normal TSH and fT4 levels were considered euthyroid (14). The TPOAb-positive and TPOAb-negative groups consisted of patients with hypothyroidism or euthyroidism (2). Obesity was characterized by a body mass index (BMI) ≥ 25 kg/m^2^ according to the Asia-Pacific classification of BMI.

### Statistical analysis

Data were entered into a Microsoft Excel spreadsheet and subsequently analyzed using SPSS for Windows, Version 21.0 (IBM Corp., Armonk, NY, USA). Normality of quantitative variables was tested with the Kolmogorov-Smirnov and Shapiro-Wilk tests using a p value < 0.05. Continuous data are presented as median (interquartile range) values, and categorical data are presented as percentages. Comparisons between groups were done using the Mann-Whitney test for continuous variables and the chi-square test for categorical variables. The significance level was set at 5%. Multivariate logistic regression analysis was carried out to identify the predictors of TPOAb positivity. Spearman’s correlation was used to evaluate correlations in data with nonparametric distribution. Receiver operating characteristic (ROC) analysis was done to assess the diagnostic accuracy of the E2/T ratio in predicting TPOAb positivity by plotting sensitivity on the Y axis as a function of [1-specificity] on the X axis.

## RESULTS

The study included 115 male patients with a median age of 34 years (Table 1). A TPOAb positivity was determined by a serum TPOAb value ≥ 35 IU/mL. The patients were divided into two groups based on the presence or absence of TPOAb (positive or negative). Individuals with positive TPOAbs, compared with those with negative TPOAbs, had a higher prevalence of goiter (88%) and obesity (61%) and higher levels of total cholesterol, triglycerides, and low-density lipoprotein (LDL) cholesterol (Table 1).

**Table 1:** Baseline characteristics of the patients with positive and negative thyroid peroxidase antibodies

Characteristics	Positive TPOAbs	Negative TPOAbs	P values
Number of patients	78	37	N/A
Age – years	34 (28-40)	34 (27-42)	0.525
BMI – kg/m^2^	24.8 (23-26.3)	23.0 (22-25.3)	0.008
Waist circumference – cm	94 (90-102)	90 (88-100)	0.230
Obesity (BMI ≥ 25 kg/m^2^) – n (%)	38 (48.7)	10 (27)	0.028*
Current smoker – n (%)	39 (50.0)	14 (37.8)	0.222
Alcohol consumption	18 (23.1)	5 (18.9)	0.614
Family history of hypothyroidism – n (%)	31 (39.7)	8 (21.6)	0.055
Hypothyroidism – n (%)	69 (88.5)	11 (29.7)	<0.001
Goiter	48 (61.5)	9 (24.3)	<0.001
Total cholesterol – mg/dL	183 (166-198)	170 (156-182)	0.006
LDL cholesterol – mg/dL	102 (88-117)	87 (80.5-98)	<0.001
Triglycerides – mg/dL	180 (154.2-201.2)	156 (137-180)	0.005
HDL cholesterol – mg/dL	40 (36-43.2)	40 (36.5-48)	0.621
Fasting blood glucose – mg/dL	90 (82.5-100)	88 (80-95)	0.352
Fasting insulin – mIU/mL	6.85 (4.4-9.8)	5.6 (3.2-7.8)	0.052
25-hydroxyvitamin D3 – ng/mL	24.2 (18-41.2)	34.0 (25-43.5)	0.072
Vitamin D < 20 ng/mL	20 (25.6)	7 (18.9)	0.427
Free thyroxine (fT4) – ng/dL	0.9 (0.7-1.0)	1.2 (1.0-1.4)	<0.001
Total triiodothyronine (T3) – ng/dL	102.0 (98.0-112.2)	114.0 (104.0-133)	<0.001
Thyroid-stimulating hormone (TSH) – mIU/mL	11.65 (6.5-36.6)	3.0 (2.4-5.6)	<0.001

Abbreviations: BMI, body mass index; HDL: high-density lipoprotein cholesterol; LDL, low-density lipoprotein cholesterol; n, number; N/A, not applicable; TPOAbs, thyroid peroxidase antibodies.

**Figure 1:**
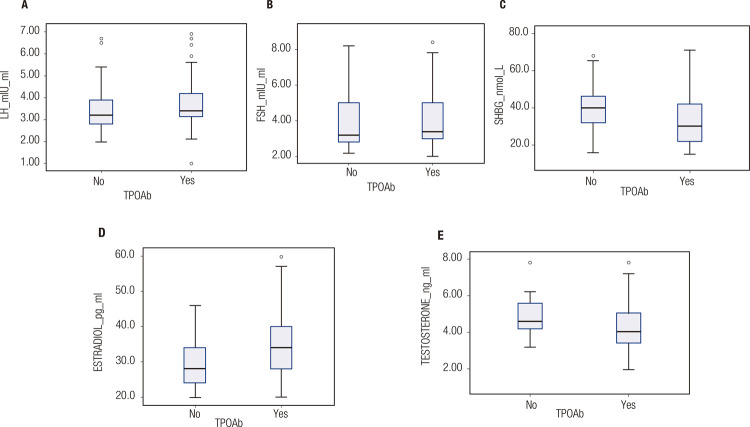
Box and whisker plots showing the median levels of (A) luteinizing hormone (LH), (B) follicle-stimulating hormone (FSH), (C) sex-hormone binding globulin (SHBG), (D) estradiol, and (E) testosterone in the groups with positive and negative thyroid peroxidase antibodies (TPOAbs).

Levels of estradiol, total testosterone, and sex-hormone binding globulin (SHBG) differed between the groups with positive and negative TPOAbs. Specifically, the median estradiol level was higher, and the median total testosterone and SHBG levels were lower in the TPOAb-positive group compared with the TPOAb-negative group (Table 2 and Figure 1). The median levels of luteinizing hormone (LH) in the TPOAb-positive and TPOAb-negative groups were, respectively, 3.4 mIU/mL (3.0-4.2 mIU/mL) and 3.2 mIU/mL (2.7-4.0 mIU/mL; p = 0.223), while the median levels of follicle-stimulating hormone (FSH) were, respectively, 3.4 mIU/mL (3.0-5.0 mIU/mL) and 3.2 mIU/mL (2.8-5.0 mIU/mL; p = 0.712), and the median E2/T ratios were, respectively, 8.00 x10^-3^ (6.66-9.77 x10^-3^) and 6.25 x10^-3^ (5.06-7.22 x10^-3^; p < 0.001).

**Table 2:** Sex hormone levels in the study population

Characteristics	Positive TPOAbs	Negative TPOAbs	P values
Estradiol – pg/mL	34.0 (28.0-40.0)	28.0 (24.0-34.0)	0.021
Testosterone – ng/mL	4.05 (3.4-5.0)	4.6 (4.2-5.6)	0.002
E2/T	8.0 (6.6-9.7) x10^-3^	6.25 (5.0-7.2) x10^-3^	<0.001
SHBG – nmol/ L	30.0 (22.0-42.2)	40.0 (32.0-46.0)	0.006
LH – mIU/mL	3.4 (3.0-4.2)	3.2 (2.7-4.0)	0.223
FSH – mIU/mL	3.4 (3.0-5.0)	3.2 (2.8-5.0)	0.712

*Data are expressed as median (interquartile range). Abbreviations: E2/T, estradiol-to-testosterone ratio; FSH, follicle-stimulating hormone; LH, luteinizing hormone; SHBG, sex-hormone binding globulin.

**Figure 2:**
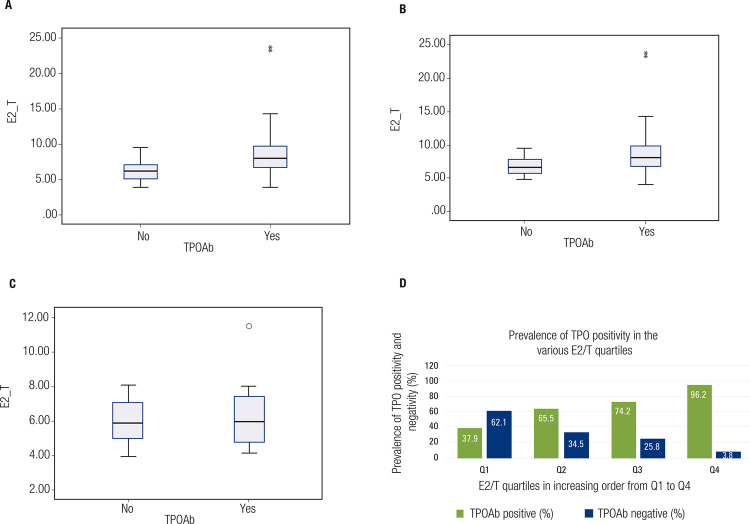
Box and whisker plots showing the median estradiol-to-testosterone (E2/T) ratio in the groups with positive and negative thyroid peroxidase antibodies (TPOAbs). (A) Overall cohort; (B) patients with hypothyroidism; (C) euthyroid patients. The bar graph in (D) shows the prevalence of positive TPOAbs based on E2/T ratio quartiles.

In subgroup analysis including only patients with hypothyroidism (n = 80), the median E2/T ratio was higher in the TPOAb-positive group (8.12 [6.80-9.95 x10^-3^]) than in the TPOAb-negative group (6.52 [5.71-8.09] x10^-3^; p = 0.016) (Figure 2B). When a similar analysis included only euthyroid individuals, no significant (p = 0.970) difference in median E2/T ratios was observed between the TPOAb-positive and TPOAb-negative groups (Figure 2C). The prevalence of TPOAb positivity increased with the increase in E2/T ratio quartiles, from 37.9% in the lowest quartile to 96.2% in the highest quartile (p value for trend across all quartiles < 0.001) (Figure 2D).

On multivariate analysis adjusted for the presence of hypothyroidism, BMI, and levels of SHBG, vitamin D3, and LDL cholesterol, the E2/T ratio emerged as an independent predictor of TPOAb positivity (odds ratio 1.376, 95% CI 1.020-1.856, p = 0.037) (Table 3). Hypothyroidism and LDL levels also emerged as independent predictors of TPOAb positivity (Table 3).

**Table 3:** Multivariate logistic regression analysis of the positivity of thyroid peroxidase antibodies adjusted for different variables

Variables	B	SE	Wald test	df	P values	OR	95% confidence interval	
							Lower	Upper
Body mass index	.046	.097	.227	1	.634	1.047	.866	1.267
Hypothyroidism	2.193	.574	14.578	1	.000	8.963	2.908	27.632
E2/T	.319	.153	4.367	1	.037	1.376	1.020	1.856
SHBG	-.029	.022	1.709	1	.191	.972	.931	1.014
Vitamin D3	.008	.019	.170	1	.681	1.008	.971	1.047
LDL cholesterol	.047	.019	6.044	1	.014	1.048	1.010	1.088
Constant	-7.690	3.249	5.601	1	.018	.000		

Abbreviations: B, intercept; df, degrees of freedom; E2/T, estradiol-to-testosterone ratio; LDL, low-density lipoprotein cholesterol; OR, odds ratios; SE, standard error; SHBG, sex-hormone binding globulin.

**Figure 3:**
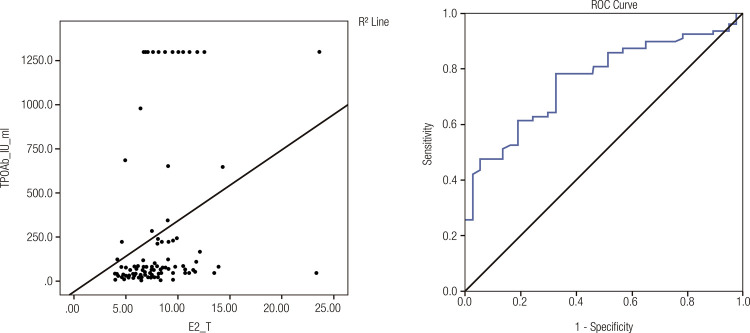
Scatterplots showing the relationship between estradiol-to-testosterone (E2/T) ratios (x10-3) and thyroid peroxidase antibodies (TPOAbs). Spearman’s correlation coefficient = 0.443. The image on the right shows a receiver operating characteristic (ROC) curve of the E2/T ratio identifying TPOAb-positive subjects (the blue line depicts the ability of the E2/T ratio in differentiating between patients with and without TPOAb. The black line is a reference line with an area under the curve of 0.5, indicating no discriminatory value).

A moderate positive correlation was observed between the E2/T ratio and TPOAb level (r = 0.443; p < 0.001). An E2/T ratio cutoff value of 6.565 x10^-3^ demonstrated the best diagnostic accuracy, with a sensitivity of 78.2%, specificity of 67.6%, and an area under the curve of 0.761 (95 % CI 0.674-0.849) (Figure 3).

## DISCUSSION

Our study evaluated the relationship between the E2/T ratio and TPOAb positivity in male patients with treatment-naïve primary hypothyroidism or euthyroidism. The results provide insight into considering the E2/T ratio as an independent predictor of thyroid disorders. The median E2/T ratio was significantly higher in the TPOAb-positive group compared with the TPOAb-negative group. We chose to analyze TPOAb as a marker of autoimmunity over alternative autoantigens (*e.g.*, TgAb) due to the higher titers associated with TPOAb and its demonstrated efficacy as a predictor of thyroid dysfunction (12,15). Since studies have shown that TPOAb titers decrease by 10%-90% after 6-24 months of levothyroxine therapy, the present study included only patients with treatment-naïve primary hypothyroidism or euthyroidism to minimize the occurrence of changes associated with hypothyroidism treatment (16-20).

In the present study, the TPOAb positivity rate was 68%, and most (88%) patients had hypothyroidism. Notably, TPOAb positivity in the general population in India ranges from 13.3% to 25.8%, with these antibodies accounting for 90% of all circulating antibodies in patients with hypothyroidism (21,22). The high prevalence of TPOAb positivity in the present study could be due to the strict inclusion criteria applied, in which only patients with signs and symptoms suggestive of hypothyroidism were included.

Previous studies have shown no significant association between TPOAb positivity and age, cigarette smoking, fasting plasma glucose, vitamin D deficiency, or family history of hypothyroidism (23-26). In the present study, the prevalence of goiter was greater in patients with TPOAb positivity. Also, a significantly greater proportion of patients with high BMI belonged to the TPOAb-positive group. In a meta-analysis of 22 studies published in 2018, obesity correlated with TPOAb positivity (risk ratio 1.93, 95% CI 1.31-2.85, p = 0.001) (27). The mechanisms linking obesity and autoimmune disorders remain unclear. Factors associated with adipokines, particularly leptin-mediated immune and inflammatory responses, contribute to the increased production of TPOAbs by shifting the balance toward a T helper 1 (Th1) cell phenotype and inhibiting the function of regulatory T (Treg) cells (28). Similar observations have been reported in other studies (29,30) showing that patients with positive TPOAbs compared with those with negative TPOAbs have higher total cholesterol (p = 0.006), LDL cholesterol (p < 0.001), and triglyceride (p = 0.005) levels but comparable high-density lipoprotein (HDL) cholesterol levels (p = 0.621). In the present study, LDL cholesterol level emerged as an independent predictor of TPOAb positivity, even after adjustments for BMI and presence of hypothyroidism.

The median SHBG level was lower in the TPOAb-positive group compared with the TPOAb-negative group (p = 0.006). Several studies have documented low SHBG levels in low-grade chronic inflammatory diseases involving cytokine changes, including TNF-α, IL-1β, and adiponectin. Additionally, a negative correlation between SHBG and leptin was demonstrated in a study by Gomez and cols. (31-33). In the present study, SHBG level was not an independent predictor of TPOAb positivity on multivariate logistic regression analysis (OR 0.972, 95% CI 0.031-1.014, p = 0.191). The median levels of LH and FSH were comparable between the groups with positive and negative TPOAbs.

The median estradiol level was significantly higher, and the median testosterone levels were significantly lower in the TPOAb-positive group. This finding is aligned with the results from the SPECT-China study (11). In a study by Chailurkit and cols., serum estradiol levels were independently associated with thyroid receptor antibodies (OR 1.17, 95% CI 1.11-1.23, p < 0.001) (10). Estrogen modulates the growth of thyroid tissue by interacting with its receptor, although estrogen administration does not alter the profile of thyroid hormones, according to some studies (10,34).

We found a higher median E2/T ratio in the TPOAb-positive compared with the TPOAb-negative group (8.00 [6.66-9.77] x 10^-3^ versus 6.25 [5.06-7.22] x 10^-3^, respectively; p < 0.001). The mean E2/T ratio in the SPECT-China study was 7.19 ± 10.30 in the TPOAb/TgAb (-) group, 7.91 ± 8.03 in the TPOAb/TgAb (+) group, and 8.78 ± 11.26 in the TPOAb/TgAb (+) and ultrasound (US) (+) group (p < 0.001) (11).

It can be speculated that the TPOAb-positive group had a greater number of patients with hypothyroidism with decreased SHBG levels, leading to reduced testosterone and estradiol levels, thus affecting the E2/T ratio. However, this is less likely to have happened in the subgroup analysis of patients with hypothyroidism, in which the median E2/T ratio was higher in the TPOAb-positive compared with the TPOAb-negative subgroup. Further analysis revealed that the percentage of TPOAb positivity increased from 37.9% in the lowest E2/T ratio quartile to 96.2% in the highest E2/T ratio quartile, with a significant (p < 0.001) trend across all quartiles.

The E2/T ratio remained an independent predictor of TPOAb positivity (p = 0.037) even after adjustment for BMI, presence of hypothyroidism, and levels of SHBG, vitamin D3, and LDL cholesterol. Hypothyroidism remained the strongest predictor of TPOAb positivity (OR 1.37, 95% CI 2.9-27.6). The SPECT-China study also presented similar results, in which increased E2/T ratios were associated with an increased risk of TPO/TgAb (+), as well as TPO/TgAb (+) and US (+) (11). In the present study, there was a moderately positive correlation (r = 0.443, p < 0.001) between E2/T ratio and TPOAb positivity (35). The E2/T ratio cutoff value of 6.565 x10^-3^ demonstrated the best diagnostic accuracy for identifying TPOAb positivity, with a sensitivity of 78.2% and specificity of 67.6%.

### Limitations of the study

Our study was conducted at a single center and included a limited sample size. The levels of sex hormones were measured on a single occasion. The association between the E2/T ratio and markers of autoimmunity may not imply causation. Furthermore, the reverse effect (hypothyroidism leading to E2/T ratio changes) should be excluded.

In conclusion, our study focused on the role of altered sex hormone levels contributing to the pathogenesis of AITDs. The results emphasized the practicality of the E2/T ratio as a predictor of thyroid disorder. Given the ever-increasing prevalence of AITDs globally and the widespread use of endocrine-disrupting chemicals, our findings highlight the role of sex hormones in immune dysfunction and their potential translation into effective treatment strategies for thyroid disorders.
